# 

**Published:** 1852-05

**Authors:** 


### CONTENTS.

ORIGINAL COMMUNICATIONS :— PAGE.

ART. 1.—Remarks on the Nature and Cause of Cancerous Disease, and the indications for its cure, by H. A. Johnson, M.D., . . . . . . 1
ART. 2.—Case of Hypertrophy and extensive disease of the right and absence of the left kidney, by W. P. Golliday, M.D., . . . . . . 9
ART. 3.—Cases of Cancrum Oris, by Charles Brackett, M.D., . . 11
ART. 4.—Morning Sickness of Pregnancy, by Seely Brownell, M.D., . . 12
ART. 5.—Chloroform applied locally in fractures, by H. N. Hurlbut, M.D., 13
ART. 6.—Treatment of Milk Sickness, by C. W. Davis, M.D., . . 13
ART. 7.—Dysentery, by Waters, . . . . . . . . . . 14

SELECTIONS :—

Report of Cases of Typhoid Fever and Typhoid Pneumonia, treated principally with Veratrum Viride, . . . . . . . . . 15
Cases of Delirium Tremens, successfully treated by the administration of Chloroform, . . . . . . . . . . . . . . . 20
Report of three Cases in which Lactation was reproduced by the application of the child to the breast, . . . . . . . . . . 22
Case of a Large Sub-Cutaneous Nævus Cured by Vaccination, . . . . 24
Cold water in Puerperal Convulsions, . . . . . . . . . 25
Causes of Albuminous Urine, . . . . . . . . . . . 26
Treatment of Asthenic Dropsy, by the preparations of Nux Vomica, . . 28
Iodine Injections into the Peritoneum in Ascites, . . . . . . 28
Substitutes for Mercurials in the Treatment of Syphilis. . . . . . 29
The Itch Cured in two hours, . . . . . . . . . . . 30
Rules for Bleeding in Pneumonia, . . . . . . . . . . 30

EDITORIAL :—

To the Profession, . . . . . . . . . . . . . . 31
Apology, . . . . . . . . . . . . . . . . . 32
Ninth Annual Report of the Managers of the State Lunatic Asylum of the State of New York, . . . . . . . . . . . . . . 32
Illinois State Medical Society, . . . . . . . . . . . 33
Bird on Urinary Deposits,—Frick on Renal Diseases, . . . . . 34
The Liver and its Diseases, . . . . . . . . . . . 40
The Meeting of the American Medical Association at Richmond, . . . 43
Formula, . . . . . . . . . . . . . . . . . 46
Common Salt in Scarlet Fever, . . . . . . . . . . 46
Medical Statistics, . . . . . . . . . . . . . . 47
Canada Medical Journal, . . . . . . . . . . . . 47
New Orleans Medical Register, . . . . . . . . . . . 48
Works Received, . . . . . . . . . . . . . . . 48

